# First trimester fetal echocardiography limitations and its expected clinical values

**DOI:** 10.1186/s43044-020-00049-1

**Published:** 2020-04-07

**Authors:** Heba Kamel, Amr Yehia

**Affiliations:** 1 Congenital and Structural Heart Disease Unit, Department of Cardiology, Faculty of Medicine, Ain Shams University Hospital, Abbassya, Cairo, Egypt; 2 Department of Obstetrics and Gynecology, Faculty of Medicine, Ain Shams University, Cairo, Egypt

**Keywords:** Fetal echocardiography, Color Doppler, 2D

## Abstract

**Background:**

Fetal cardiac activity could be observed between 6th and 7th gestational weeks, early performance of fetal echocardiography could be implemented to screen for fetal heart disease. The effectiveness of early first trimester fetal echocardiography has not been adequately investigated, especially with modern sonographic technological advances. The purpose of the study is to evaluate the capability to visualize fetal cardiac structures within the first trimester as early as 10th gestational weeks and to elucidate the value of using color Doppler in visualization of cardiac structures within early gestation. A prospective clinical trial conducted on 150 study subjects, 44 of them were twin gestations. Cases were fully assessed by fetal echocardiographic examination from 10th gestational week to 13 gestational weeks in a sequential manner weekly. The research study was conducted at cardiology department fetal unit in one of the tertiary hospitals.

**Results:**

Four chamber view was mostly visualized from 12 gestational weeks, whereas cardiac axis was fully visualized in all cases from 12 gestational weeks; on the other hand, IVC assessment by 2D was satisfactorily visualized in 78.26% of cases and by color Doppler in 82.61% of cases at 13 gestational weeks, pulmonary veins were visualized in 21.74% of cases by 2D and 43.5% of cases by color Doppler at 13 gestational weeks, and interestingly, ventricular inflows were satisfactorily visualized in almost all cases from 10th gestational weeks.

**Conclusions:**

First trimester fetal echo is an outstanding enhancement in management pathways of cases susceptible to have fetal cardiac abnormalities permitting early detection of structural cardiac anomalies triggering a cascade of scanning for extra cardiac anomalies to aid in evaluation and assessment of the best management course for those affected cases.

## Background

Fetal echocardiographic scanning has shown recent great advances in which it was routinely practiced from 20 to 24 gestational weeks to determine the presence of congenital heart diseases [1, 2].

However recently, there is a growing research interest to develop an earlier approach to diagnose major structural cardiac anomalies at earlier gestations within the first trimester that is aided by the upgrading in the frequency of sonographic waves produced from abdominal and vaginal probes and other image enhancing features integrated to display the fetal structures in a detailed manner as early as 8 gestational weeks [3, 4].

Congenital heart diseases are considered challenging cases as they require multidisciplinary management approach by integrated efforts between the sonographer, obstetrician, and the neonatologists; therefore, early diagnosis could further enhance and improve clinical outcomes by permitting establishment of suitable and safe circumstances during delivery [5, 6].

Transposition of great arteries and hypoplastic chambers among other cardiac anomalies are responsible for chief mortalities among neonates particularly when diagnosed after delivery; therefore, the art of fetal echocardiography and systematic scanning of the fetal developing cardiac structures with evaluation of blood flow indices and parameters is considered a lifesaving practice in which it permits to secure the affected fetuses to be delivered in the presence of proper NICU capabilities and permits the cardiologist to evaluate and manage the cardiac function to sufficiently prescribe the best and safest management protocol for neonates affected by cardiac disease. Even in cases that could be corrected by cardiac surgery, early intrauterine diagnosis could allow early preparation for life saving surgical approaches [7, 8].

However, it is still a developing practice to perform fetal echocardiography within first trimester and it is expected to be a commonly performed approach by enhancements available for fetal imaging among which nuchal translucency is routinely performed in first trimester scanning [7, 9].

As research efforts within this field of first trimester scanning increase, that would aid the experience and skills available to rise allowing enhanced safe clinical practice aiding in reducing mortalities among neonates [10, 11].

### Aim of the work

The aim of this study is to evaluate the capability to visualize fetal cardiac structures within the first trimester from as early as 10th gestational weeks and to elucidate the value of using color Doppler in visualization of cardiac structures within early gestation.

## Methods

A prospective clinical trial conducted on 150 study subjects, 44 of them were twin gestations in which they were fully assessed by fetal echocardiographic examination from the 10th gestational week to 13 gestational week in a sequential manner weekly, then fetal echo was repeated at the 18th gestational week to compare and contrast the obtained results to those obtained at 10th till 13th gestational weeks. The research study was conducted at cardiology department fetal unit in one of the tertiary hospitals.

The sonographic machines used were GE Vivid E9 and GE vivid s5. Transabdominal scanning, either a 4.5-MHz phased array transducer (M5S) or a 7-MHz phased array transducer (6S) were used. Gestational ages have been obtained using the LMP or early sonographic scan if available. Crown-rump length (CRL) was attained from all recruited study subjects.

### Fetal echocardiographic methodology

Using the transabdominal imaging, the transducer has been situated just above the symphysis pubis, plain of imaging was oriented in a manner to be behind the pubic bone, the gain was adjusted to be most of the time of examination to be above 80 Hz. For visualization of the fetal cardiac anatomical structures (e.g., four chambers, great vessels, and arches) four-chamber imaging, involving the mitral and tricuspid valves, both great arteries with display of great vessels crossing, aortic and ductal arches, and IVC and pulmonary vein, have been approached in all fetuses. The three-vessel imaging permitted assessment of arch position, size, and patency besides relationship to the trachea. Sagittal plane evaluation of the arches supplied valuable sonographic data and findings as regards the arch size and flow.

Furthermore, 2D and color Doppler imaging have been conducted in all research study subjects; both thermal index (TI) and mechanical index (MI) were recorded.

### Statistical analysis

Data were collected, revised, coded, and entered to the Statistical Package for Social Science (IBM SPSS) version 23. Qualitative variables were presented as number and percentages, and comparison between groups regarding qualitative data was done by using chi-square test and/or Fisher exact test when the expected count in any cell found less than 5. The confidence interval was set to 95% and the margin of error accepted was set to 5%. So, the *p* value was considered significant at the level of < 0.05.

## Results

Table 1 reveals and displays that the mean maternal age was +/−SD = 26.7 ± 5.6 years, mean maternal weight was 73.54 +/− 12.36, and mean body mass index (BMI) was 24.63 +/− 4.75; gestational age at study entry was mean ± SD = 9.5 ± 0.4 gestational weeks, as regards parity, 50 cases were PG representing 33.3% of cases, 75 cases were P1 representing 50.0% of cases, and 25 cases were P2 representing 16.7%; number of singleton gestations = 106 fetuses representing 70.7% of total number of fetuses, number of twins = 44 fetuses representing 29.3% of fetuses, and total number of fetuses = 194.

**Table 1:** Demographic data of the studied cases

	No. = 150
Maternal age, mean ± SD	26.7 ± 5.6
GA at study entry, mean ± SD	9.5 ± 0.4
Weight, mean+/−SD Body mass index, mean+/−SD	73.54 +/− 12.36 24.63 +/− 4.75
Parity	
PG	50 (33.3%)
1	75 (50.0%)
2	25 (16.7%)
Number of babies	
Single	106 (70.7%)
Twin	44 (29.3%)
Total number of babies	194 (100.0%)

**Fig. 1:**
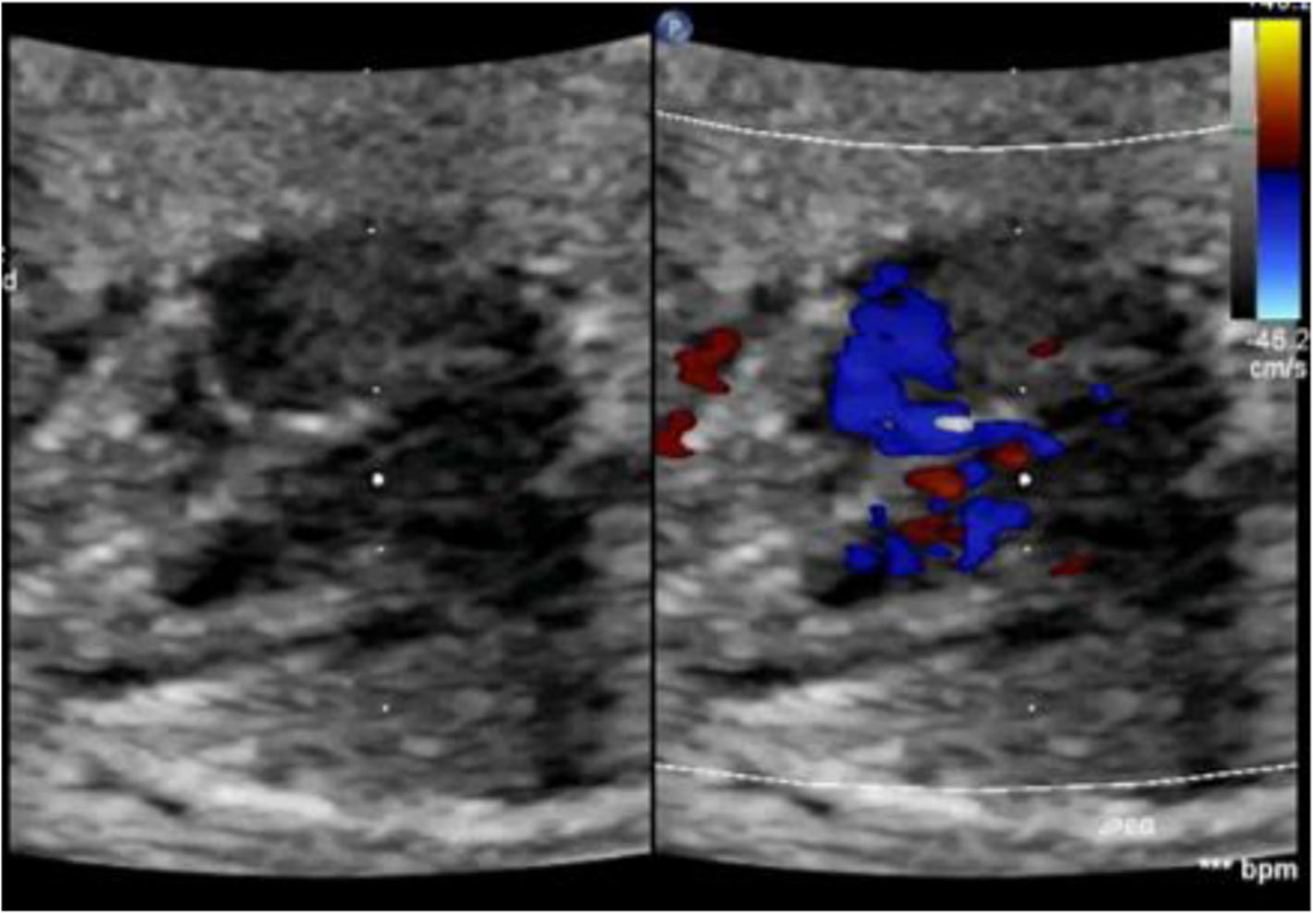
Four chamber view both by 2D and color Doppler imaging in a case 12-week gestation

**Fig. 2:**
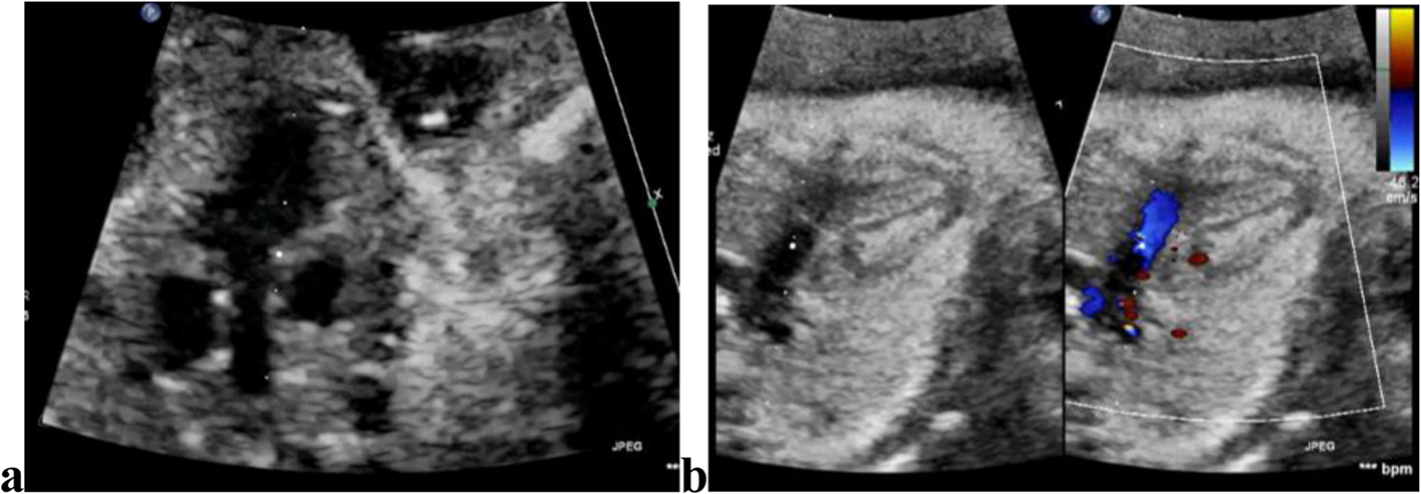
**a** Shows left ventricle outflow tract view by 2D imaging in a case 13-week gestation. **b** Shows right ventricle outflow tract by both 2D and color Doppler imaging in a case 11-week gestation

Table 2 reveals and displays fetal echocardiographic findings observed at different gestational ages investigated by 2D; four chamber view was observed in 184 (94.8%) fetuses at 10th gestational weeks, in 188 fetuses at 11th gestational weeks (96.9%), at 12th, 13th, 18th gestational weeks in 194 fetuses (100.0%) (Fig. 1). Cardiac axis was observed in 144 fetuses (74.2%) at 10th gestational weeks, 186 fetuses (95.9%) at 11th gestational weeks, 194 fetuses (100.0%) at 12th,13th ,18th gestational weeks. Ventricular inflows were observed 189 fetuses (97.4%) at 10th gestational weeks, 192 fetuses (99.0%) at 11th gestational weeks, 194 fetuses (100.0%) at 12th, 13th, 18th gestational weeks (Fig. 2). Thermal index at 10th, 11th, 12th, 13th, 18th gestational weeks =0.29 ± 0.1, 0.31 ± 0.11, 0.23 ± 0.09, 0.22 ± 0.10, 0.25 ± 0.1 consecutively. Mechanical index at 10th, 11th, 12th, 13th, 18th gestational weeks = 0.87 ± 0.15, 0.92 ± 0.21, 1.1 ± 0.27, 1.2 ± 0.28, 0.7 ± 0.2 consecutively. Average ultrasound time (min) at 10th, 11th, 12th, 13th, 18th gestational weeks = 22.5 ± 12.27, 29.3 ± 10.27, 28.7 ± 9.45, 31.2 ± 9.25, 32.4 ± 8.75 min consecutively.

**Table 2:** Fetal echo cardiac structures observed by echocardiography at different gestational ages investigated by 2D

	Gestational age (weeks)				
	10th	11th	12th	13th	18th
Four chamber view	184 (94.8%)	188 (96.9%)	194 (100.0%)	194 (100.0%)	194 (100.0%)
Cardiac axis	144 (74.2%)	186 (95.9%)	194 (100.0%)	194 (100.0%)	194 (100.0%)
Ventricular inflow	189 (97.4%)	192 (99.0%)	194 (100.0%)	194 (100.0%)	194 (100.0%)
TI	0.29 ± 0.1	0.31 ± 0.11	0.23 ± 0.09	0.22 ± 0.10	0.25 ± 0.1
MI	0.87 ± 0.15	0.92 ± 0.21	1.1 ± 0.27	1.2 ± 0.28	0.7 ± 0.2
Average ultrasound time	22.5 ± 12.27	29.3 ± 10.27	28.7 ± 9.45	31.2 ± 9.25	32.4 ± 8.75

**Fig. 3:**
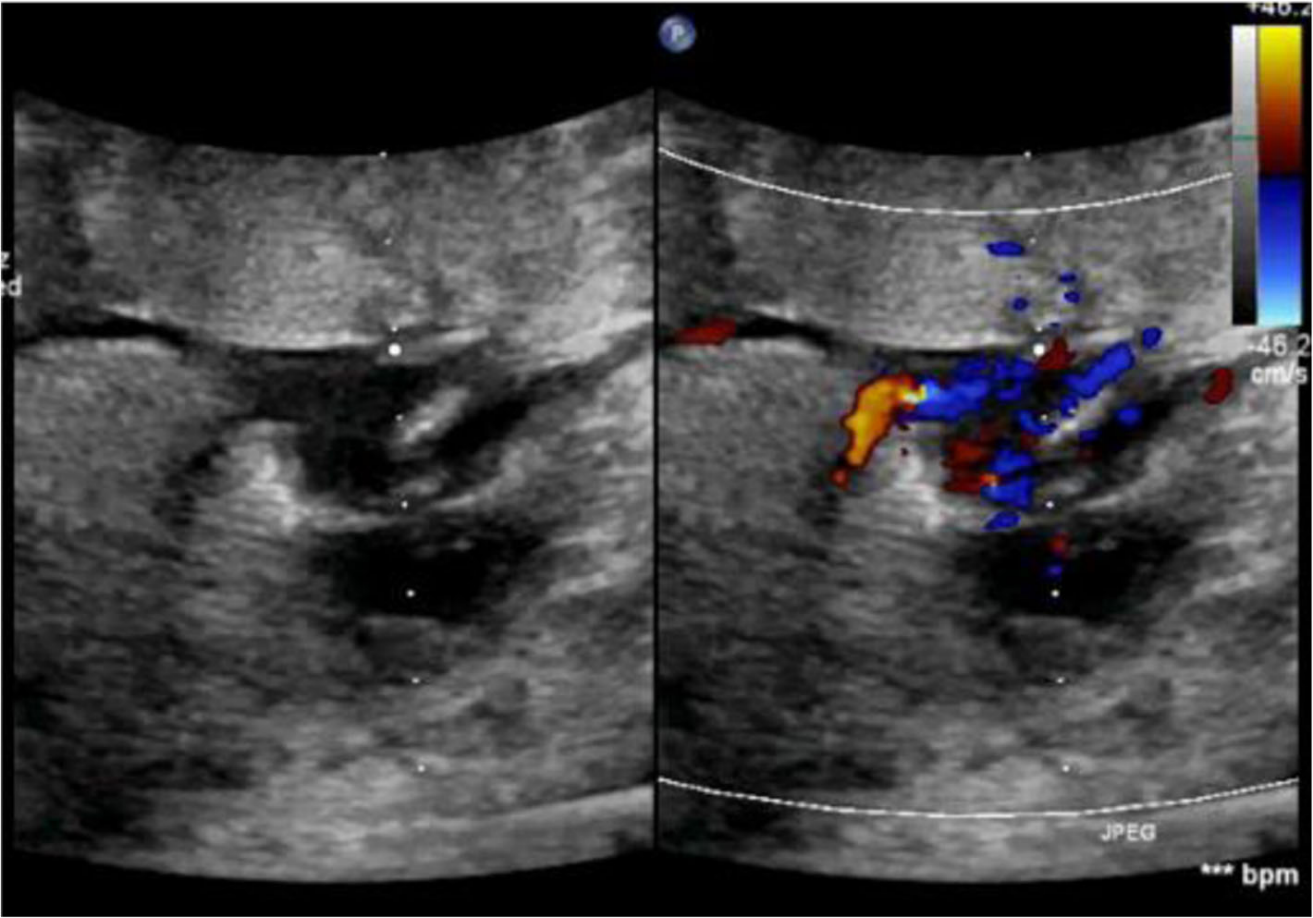
Normal systemic veno-atrial connection by both 2D and color Doppler imaging in a case 12-week gestation

**Fig. 4:**
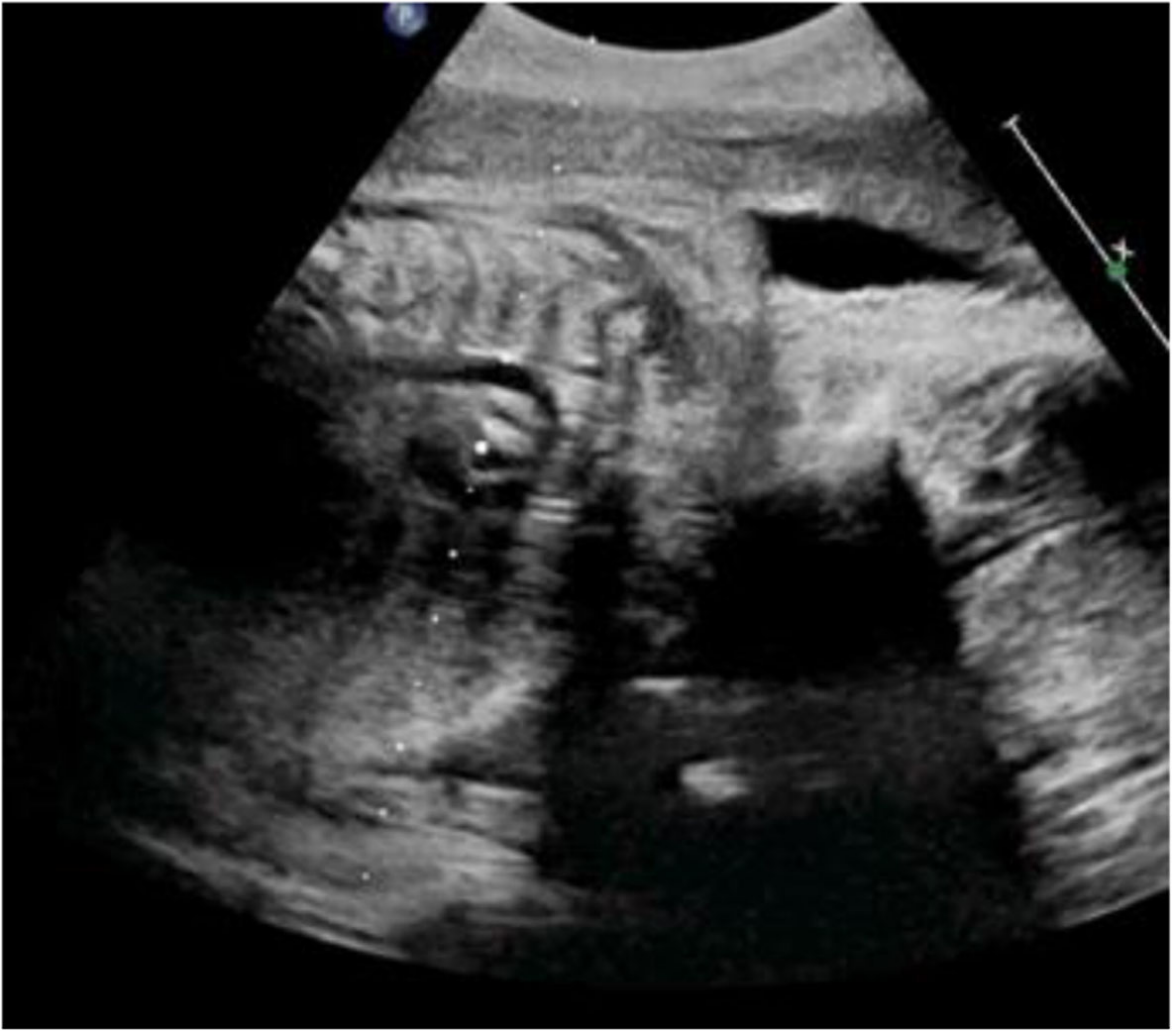
2D imaging of the aortic arch in a case 13-week gestation

Table 3 reveals and displays fetal echocardiographic findings observed at different gestational ages investigated by 2D and color Doppler; IVC was observed by 2D in 25 fetuses (12.9%) at 10th gestational weeks, 87 (44.8%) at 11th gestational weeks, 91 fetuses (46.9%) at 12th gestational weeks, 155 fetuses (79.9%) at 13th gestational weeks, and 179 fetuses (92.3%) at 18th gestational weeks, whereas IVC was observed by color Doppler in 171 fetuses (88.1%) at 10th gestational weeks, 179 fetuses (92.3%) at 11th gestational weeks, 180 fetuses (92.8%) at 12th gestational weeks, 182 fetuses (93.8%) at 13th gestational weeks, and 190 fetuses (97.9%) at 18th gestational weeks (Fig. 3). Color Doppler in comparison to 2D in observation of IVC, pulmonary veins, both left and right outflow tracts, crossing of great arteries, aortic arch (Fig. 4) and ductal arch was statistically significant and more superior in detectability by 2D since most *p* values < 0.001. At 13th gestational weeks, color Doppler did not show statistically significant difference in comparison to performance of echocardiography at 18th gestational weeks (Table 3).

**Table 3:** Fetal cardiac structures observed and investigated by echocardiographic examination using 2D and color Doppler at various gestational ages

	2D	*p* value^*^	Color Doppler	*p* value^*^	*p* value^‡^
IVC					
10th w	25 (12.9%)	**< 0.001**	171 (88.1%)	**< 0.001**	**< 0.001**
11th w	87 (44.8%)	**< 0.001**	179 (92.3%)	**0.010**	**< 0.001**
12th w	91 (46.9%)	**< 0.001**	180 (92.8%)	**0.016**	**< 0.001**
13th w	155 (79.9%)	**< 0.001**	182 (93.8%)	**0.041**	**< 0.001**
18th w	179 (92.3%)	–	190 (97.9%)	–	**0.009**
Pulmonary veins					
10th w	0 (0.0%)	**< 0.001**	0 (0.0%)	**< 0.001**	1.000
11th w	0 (0.0%)	**< 0.001**	6 (3.1%)	**< 0.001**	**0.013**
12th w	6 (3.1%)	**< 0.001**	18 (9.3%)	**< 0.001**	**0.011**
13th w	49 (25.3%)	0.306	89 (45.9%)	**0.025**	**< 0.001**
18th w	58 (29.9%)	–	111 (57.2%)	–	**< 0.001**
Both left and right outflow tracts					
10th w	45 (23.2%)	**< 0.001**	128 (66.0%)	**< 0.001**	**< 0.001**
11th w	146 (75.3%)	**< 0.001**	179 (92.3%)	**< 0.001**	**< 0.001**
12th w	150 (77.3%)	**< 0.001**	185 (95.4%)	**0.004**	**< 0.001**
13th w	186 (95.9%)	**0.004**	191 (98.5%)	0.652	0.126
18th w	194 (100.0%)	–	194 (100.0%)	–	1.000
Crossing greater arteries					
10th w	44 (22.7%)	**< 0.001**	103 (53.1%)	**< 0.001**	**< 0.001**
11th w	129 (66.5%)	**< 0.001**	173 (89.2%)	**< 0.001**	**< 0.001**
12th w	140 (72.2%)	**< 0.001**	178 (91.8%)	**0.007**	**< 0.001**
13th w	177 (91.2%)	**< 0.001**	187 (96.4%)	0.092	**0.035**
18th w	189 (97.4%)	–	192 (99.0%)	–	0.252
Aortic arch					
10th w	54 (27.8%)	**< 0.001**	120 (61.9%)	**< 0.001**	**< 0.001**
11th w	124 (63.9%)	**< 0.001**	160 (82.5%)	**< 0.001**	**< 0.001**
12th w	142 (73.2%)	**< 0.001**	182 (93.8%)	**0.041**	**< 0.001**
13th w	179 (92.3%)	**< 0.001**	187 (96.4%)	0.358	0.079
18th w	189 (97.4%)	–	190 (97.9%)	–	0.735
Ductal arch					
10th w	54 (27.8%)	**< 0.001**	120 (61.9%)	**< 0.001**	**< 0.001**
11th w	127 (65.5%)	**< 0.001**	167 (86.1%)	**< 0.001**	**< 0.001**
12th w	151 (77.8%)	**< 0.001**	185 (95.4%)	**0.032**	**< 0.001**
13th w	182 (93.8%)	**< 0.001**	186 (95.9%)	0.055	0.358
18th w	190 (97.9%)	–	192 (99.0%)	–	0.410

Comparison done using chi-square test

Bold indicates significant

‡*p* value in comparison between 2D and color Doppler

*p value in comparison with gestational age at 18th week

## Discussion

First trimester echocardiographic screening is an increasingly common practice performed by skilled professionals; however, it requires more skills and experience to be acquired since the degree of visualization of different cardiac structures vary in a considerable fashion and rely on operator and machine related factors. First trimester screening for cardiac anomalies could be used as an adjunct to routine anomaly scanning performed from 18 to 22 gestational weeks [1, 2].

Fetal cardiac development could be affected by chronic medical disorders such as DM and could be observed more frequently in families with history of consanguinity and congenital heart disease. So, there is an increasing trend as regards the indications for early first trimester fetal echocardiography aided with advanced technology [13, 15].

A cornerstone issue that allows the parents to gain benefit from early diagnosis is to have time to make informed decisions concerning their pregnancy [16].

Furthermore, congenital defects observed within the first gestational trimester could be assessed for their developmental progress and possible prognosis that could be implemented in family discussion and counseling that denotes that more minor fetal cardiac anomalies diagnosed early could be followed up throughout gestation to determine the degree of progression providing clinical opportunities for earlier interventional management and provides a clue for the prognosis post-natal period [1, 3].

The current research study findings have interestingly revealed that color Doppler in comparison to 2D in observation of IVC, pulmonary veins, left and right outflow tracts, crossing of great arteries, aortic arch, ductal arch was statistically significant and more superior in detectability since most *p* values < 0.001. Denoting the privilege of color Doppler in detectability of fetal cardiac vessels at early gestational ages that permits detailed fetal cardiac scanning and increases the detectability of cardiac vascular anomalies. That was in agreement with other research group that showed improvement in detection rate with color Doppler than with 2D imaging for different structures as for example the IVC which was visualized by 2D imaging in only 4% in the eighth week, increasing to 13% by the 10th week and 80% by the 13th week where CD improved visualization of the inferior vena cava at earlier GAs to > 80% from 10 weeks [9]. Also, Wener et al. showed that visualization of the 4CV, outflow tract views, and three-vessel view was possible in 90% of fetuses at 12 to 14 weeks [14]. In our study at 13th gestational weeks, color Doppler did not show statistically significant difference in comparison to performance of echocardiography at 18th gestational weeks. Those findings denote that most severe congenital heart anomalies and structural flow abnormalities are feasible to detect at 13th gestational weeks permitting the obstetrician and pediatric cardiologist to counsel the case about the prognosis and progress of any observed malformation.

Prior research studies more than a decade ago have revealed and displayed that full echocardiographic examination of fetal heart could be performed at satisfactory level at 10th gestational weeks in most cases; however, a prior research study similar to the current research in approach and methodology have shown that color Doppler have a cornerstone value in elucidating the detailed fetal cardiac anatomy especially outflow tracts and venous systems from 10th to 14th gestational weeks; those research findings show great harmony and similarity to the current research study findings. Privilege of early sonographic diagnosis of fetal cardiac pathology also could be a trigger for detailed anomaly scanning for possible associated structural and chromosomal abnormalities [2, 4].

Prior research studies like the current research have mentioned that the best timing for fetal heart evaluation in a complete manner within the first trimester is between 12 + 0 and 13 + 6 gestational weeks [7].

Hutchinson and co-researchers verified the fact that to obtain a high rate of success for fetal anatomic cardiac evaluation, early fetal echo must be conducted after 11 gestational weeks [9]. On the other hand, in a smaller percentage of gestations, cardiac sonographic evaluation could be possible from 10th gestational week as sonographic researchers were in harmony with the current research study findings which have revealed that the real challenge is in the pulmonary vein evaluation in early fetal echocardiographic performance, with less than 50% successfully assessed by color Doppler even at 13 gestational weeks [10, 12].

Developmental changes in the morphology and function of the early heart need to be considered in early fetal echocardiographic performance. Since prior research groups in a similar approach and methodology have mentioned that at 10th gestational weeks, the cardiothoracic ratio is generous, the fetal cardiac axis nearly midline, with relatively large atrial chambers in comparison to the remaining cardiac mass, and a pericardial effusion is a frequent, findings interestingly research investigators have mentioned among their findings that assessment of atrioventricular valves is problematic at earlier gestational ages, especially before 12 gestational weeks. Those research findings could be justified by the fact that their thin anatomical nature besides the rapid fetal heart rate at earlier phases of intrauterine development impedes their proper sonographic resolution [8, 11].

## Conclusions

First trimester fetal echocardiography is an outstanding enhancement in management pathways of cases susceptible to have fetal cardiac problems permitting early detectability of structural cardiac anomalies triggering a cascade of scanning for extra cardiac anomalies to aid in evaluation and assessment and the best management course plan for those affected cases. Yet still second trimester scanning is the gold standard, and early screening is mainly for more understanding of the development, academic, and other many purposes. It supplies cornerstone data that could be implemented in fetal progress of cardiac pathology with available observations to discuss with the parents. However, more research efforts are required in a multicentric fashion putting in consideration racial, ethnic differences in rate of fetal cardiac development besides the need to consider medical disorders such as DM that could raise the risk of cardiac anomalies in order to have in the future a clinical algorithm that could upgrade the course of management of those category of cases.

## Data Availability

The datasets generated during and/or analyzed during the current study are available from the corresponding author on reasonable request.
